# Deep Metric Learning-Based Classification for Pavement Distress Images

**DOI:** 10.3390/s25134087

**Published:** 2025-06-30

**Authors:** Yuhui Li, Jiaqi Wang, Bo Lü, Hang Yang, Xiaotian Wu

**Affiliations:** 1 School of Physics, Northeast Normal University, Changchun 130024, China; liyuhui@nenu.edu.cn; 2 Liaoning Water Conservancy and Hydropower Survey, Design and Research Institute Co., Ltd., Liaoning 110000, China; 3 Changchun Institute of Optics, Fine Mechanics and Physics, Chinese Academy of Sciences, Changchun 130033, China; wjqdegongzuo@163.com (J.W.); lvbo@ciomp.ac.cn (B.L.); yanghang@ciomp.ac.cn (H.Y.)

**Keywords:** pavement distress detection, deep metric learning, SoftTriple loss, similarity metric, image classification

## Abstract

This study proposes a deep metric learning-based pavement distress classification method to address critical limitations in conventional approaches, including their dependency on large training datasets and inability to incrementally learn new categories. To resolve high intra-class variance and low inter-class distinction in distress images, we design a CNN head with multi-cluster centroins trained via SoftTriple loss, simultaneously maximizing inter-class separation while establishing multiple intra-class centers. An adaptive weighting strategy combining sample similarity and class priors mitigates data imbalance, while soft-label techniques reduce labeling noise by evaluating similarity against support-set exemplars. Evaluations on the UAV-PDD2023 dataset demonstrate superior performance—3.2% higher macro-recall than supervised learning, and 6.7%/8.5% improvements in macro-F1/weighted-F1 over iCaRL incremental learning—validating the method’s effectiveness for real-world road inspection scenarios with evolving distress types and limited annotation.

## 1. Introduction

The rapid advancement of global urbanization and transportation infrastructure has led to exponentially growing demands for road maintenance worldwide. Traditional pavement distress detection through manual inspection presents well-documented limitations including labor-intensive processes and inconsistent evaluation standards. While computer vision-based approaches utilizing conventional image processing techniques have demonstrated improved efficiency in road condition assessment [1,2], their performance remains heavily dependent on expert-designed features and domain-specific knowledge. Such methods often fail to maintain a robust performance when applied to previously unseen distress patterns or varying environmental conditions [3,4].

Recent years have witnessed remarkable progress in applying deep learning to pavement distress classification [5,6,7,8]. Pioneering work by Cha et al. [9] successfully integrated convolutional neural networks with sliding window techniques for high-resolution crack detection. Subsequent developments include Lei et al.’s [10] grid-based deep learning approach for crack classification and Pauly et al.’s [11] demonstration of deeper neural networks’ superior crack detection capabilities. The field further evolved with Zou et al.’s [12] DeepCrack network implementing multi-scale feature fusion and Panella et al.’s [13] hybrid method combining deep learning with traditional image processing for tunnel inspections. More recently, Li et al.’s [14] GoogLeNet-based system and Tran et al.’s [15] two-stage Mask R-CNN framework have pushed the boundaries of crack severity classification.

Despite these technological advancements, critical challenges persist in practical implementations. Current deep learning models typically require large quantities of balanced training data, which proves particularly problematic for rare distress types that are difficult to document. The models also exhibit significant performance degradation when encountering test data that deviates from the training distribution. Perhaps most crucially, conventional systems lack the capability to dynamically incorporate new distress categories after initial deployment, severely limiting their long-term utility in evolving real-world environments.

To overcome these limitations, this study introduces an innovative deep metric learning framework built upon several key technical advancements. The proposed system employs a BNInception-based [16] feature encoder trained with SoftTriple loss [17] to extract highly discriminative feature representations. A novel similarity-based classification strategy enables the effective few-shot recognition of rare distress patterns. Most importantly, the architecture supports dynamic category expansion without requiring complete model retraining.

Although recent transformer-based networks [18,19,20,21] have been applied to pavement defect detection, their role is essentially restricted to feature extraction. In fact, our BNInception backbone can be seamlessly replaced by any other feature extractor—including transformer-based ones—without altering the core of our approach. The main focus of this work is not the backbone architecture, but rather the design of an effective incremental classification mechanism for new distress classes.

Similarly, state-of-the-art incremental learners such as DER [22] and FOSTER [23] excel at mitigating catastrophic forgetting via distillation, replay, or specialized network branches, but they pay comparatively little attention to real-world challenges like high intra-class variance, ambiguous inter-class boundaries, and severe data imbalance. In contrast, our method combines SoftTriple loss with support-set based multi-centroid modeling and an adaptive weighting strategy to dynamically construct multiple class centers in feature space. This enables it to capture the complex intra-class distributions of pavement defects while enhancing inter-class separability. Moreover, since our framework does not depend on any particular backbone or heavy feature-augmentation module, it remains lightweight, broadly applicable, and well suited for deployment on resource-constrained platforms such as UAVs.

Comprehensive experimental results demonstrate the method’s superiority over conventional approaches, showing significant improvements in both detection accuracy and operational flexibility for real-world road maintenance applications.

## 2. Theoretical Foundations

### 2.1. Deep Metric Learning

Deep metric learning (DML) is a crucial subfield of machine learning that focuses on learning adaptive distance or similarity metrics in data space. Unlike traditional fixed metrics (e.g., Euclidean distance), DML learns a task-specific metric function that dynamically adjusts distance or similarity measurements based on the problem requirements. This adaptability makes DML particularly effective for handling complex data distributions and uncovering intrinsic data structures.

The primary objective of DML is to learn an embedding function that maps input samples into a metric space, where an optimized distance or similarity measure is defined. Classical metric learning methods often employ the Mahalanobis distance [24] to quantify the similarity between two feature vectors:

(1) $$\begin{array}{c} dist_{M}\left(x_{i},x_{j}\right)={\left(x_{i}-x_{j}\right)}^{T}M\left(x_{i}-x_{j}\right) \end{array}$$

where *M* is a learned positive semi-definite matrix that adaptively adjusts the distance metric based on data characteristics. This learned metric better captures the underlying relationships in the data, improving performance in downstream tasks such as classification and clustering.

The core objective of deep metric learning is to learn a metric function such that the distance between features of samples from the same class is minimized in the feature space, while the distance between features of samples from different classes is maximized—or, alternatively, to maximize the similarity among same-class features and minimize it among different-class features. This metric-based approach effectively distinguishes samples of different categories, thereby improving the accuracy and robustness of tasks such as classification and clustering.

In scenarios with sparse samples, deep metric learning can enhance model performance by leveraging similarity information between samples. Unlike traditional classification methods, deep metric learning does not require explicit category definitions; instead, it distinguishes samples by measuring their pairwise similarity through the learned metric function. As a result, pavement damage image classification methods based on deep metric learning can overcome challenges in traditional deep learning approaches, such as difficulties in sample annotation and poor generalization ability.

### 2.2. BNInception Network

The BNInception (Batch Normalization Inception) is a deep convolutional neural network architecture and a variant of the Inception series. Its primary innovation lies in the incorporation of batch normalization (BN) technology, which accelerates network convergence and enhances model performance.

**Figure 1:**
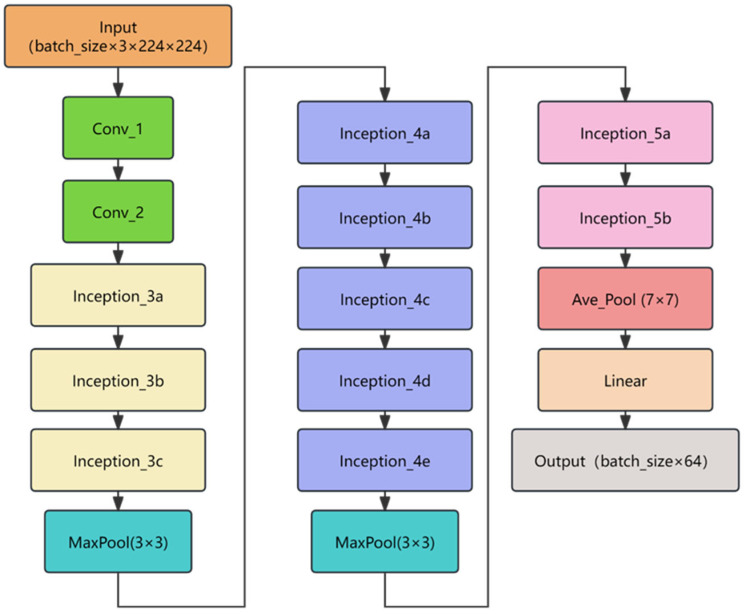
BNInception network structure.

The BNInception architecture builds upon Inception v2 and Inception v3, adopting a similar design philosophy of Inception modules. Specifically, it employs multiple parallel branches to extract features at different scales and resolutions, subsequently concatenating or aggregating these features to produce richer and more diverse representations. Additionally, BNInception introduces batch normalization layers after each convolutional layer, normalizing the outputs to stabilize training. This technique mitigates issues such as vanishing or exploding gradients, thereby improving both convergence speed and generalization capability. The network architecture of BNInception is shown in Figure 1.

BNInception exhibits three key characteristics: multi-scale feature extraction, batch normalization, and lightweight design. By utilizing convolutional kernels and pooling operations of varying sizes in parallel, the network effectively captures both local and global image features. The integration of batch normalization not only accelerates training but also enhances the model’s robustness to shifts in input data distribution. Compared to networks like Inception v3, BNInception reduces both parameter count and computational complexity, making it particularly suitable for deployment in resource-constrained environments.

In this paper, BNInception is used solely as a feature extractor to extract features of pavement distress, and this component can be replaced with any other feature extractor.

### 2.3. SoftTriple Loss

SoftTriple is a loss function designed for metric learning in classification tasks, addressing the challenge of learning discriminative feature space representations. Recognized for its simple yet effective design [25,26], SoftTriple loss consists of two key components: a classification term and a regularization term.

The core idea of SoftTriple is to define a distance metric suitable for metric learning by learning a set of class-specific centers. Unlike traditional deep metric learning methods, SoftTriple not only considers intra-class distance relationships but also incorporates inter-class relationships. Specifically, SoftTriple introduces a parameterized class center matrix and learns the distances between samples and these centers. This enables the mapping of samples onto a high-dimensional feature space where the distances between samples of the same class are minimized, and the distances between samples of different classes are maximized.

SoftTriple loss is computed as follows. Let the feature vector of an input sample be $x\in R^{d}$, with the corresponding true label y∈{1,2,…,C}. In order to better capture intra-class diversity, SoftTriple loss introduces K cluster centers for each class. Denote the k-th center of the j-th class as $c_{j,k}\in R^{d},j=1,\ldots ,C,\;k=1,\ldots ,K,$ and all centers are $L_{2}$-normalized:

(2) $$\begin{array}{c} ∥c_{j,k}{∥}_{2}=1 \end{array}$$

The cosine similarity between the sample x and each center is defined as

(3) $$\begin{array}{c} s_{j,k}=x^{⊤}c_{j,k} \end{array}$$

For each class j, compute the weight for each of the K centers using the softmax function:

(4) $$\begin{array}{c} p_{j,k}=\frac{\exp\left(\gamma s_{j,k}\right)}{\sum_{l=1}^{K}\exp\left(\gamma s_{j,l}\right)}\; \end{array}$$

where γ>0 is the temperature parameter. Consequently, the aggregated similarity of the sample to class j is given by

(5) $$\begin{array}{c} S_{j}=\sum_{k=1}^{K}p_{j,k}s_{j,k} \end{array}$$

To enhance inter-class separation, a margin m is subtracted from the aggregated similarity corresponding to the true class y. That is, define the adjusted similarity as

(6) $$\begin{array}{c} {\overset{\sim }{S}}_{j}=\left\{\begin{array}{cc} S_{j}-m, & \mathrm{if}\;j=y,\\ S_{j}, & \mathrm{otherwise}. \end{array}\right.\;\; \end{array}$$

Scale each class similarity by a factor λ>0 to obtain the logits:

(7) $$\begin{array}{c} z_{j}=\lambda {\overset{\sim }{S}}_{j},j=1,\ldots ,C \end{array}$$

Then, the classification loss is computed using the cross-entropy loss:

(8) $$\begin{array}{c} L_{\mathrm{cls}}=-\log\frac{\exp\left(z_{y}\right)}{\sum_{j=1}^{C}\exp\left(z_{j}\right)} \end{array}$$

To prevent multiple centers within the same class from being overly similar (or, in some implementations, to merge redundant sub-centers to reduce overfitting), a regularization term is introduced for the centers. After normalization, the pairwise cosine similarity between centers is

(9) $$\begin{array}{c} \cos\theta_{j,k_{1},k_{2}}=c_{j,k_{1}}^{⊤}c_{j,k_{2}} \end{array}$$

The regularization term is defined as

(10) $$L_{\mathrm{reg}}=\frac{1}{CK\left(K-1\right)}\sum_{j=1}^{C}\sum_{\begin{array}{c} k_{1},k_{2}=1\\ k_{1}\neq k_{2} \end{array}}^{K}\sqrt{2-2\cos\theta_{j,k_{1},k_{2}}+\varepsilon }$$

where ε is a small constant added for numerical stability. Combining the above components, the SoftTriple loss is defined as

(11) $$\begin{array}{c} L=L_{\mathrm{cls}}+\tau L_{\mathrm{reg}} \end{array}$$

where τ>0 is the weight hyperparameter for the regularization term.

## 3. Method

### 3.1. Problem Definition

Assuming the use of a CNN as a feature extractor, which maps the feature maps of input images to an N-dimensional vector space. In this procedure, the objective is to depict the characteristic vectors of diverse categories within this vector space in a manner that minimizes the distances between vectors belonging to the same class while maximizing the distances between vectors from different classes. Ultimately, this model generates feature vectors with N dimensions to distinguish various road surface defects in images. In order to tackle this issue, there are two crucial inquiries that need to be addressed. Firstly, it is essential to determine the suitable loss function and training methodology for effectively training this model. This involves selecting a loss function to guide how the network effectively separates feature vectors of different classes in the vector space. The choice of training approach is crucial as it determines how network parameters are updated during training to maximize the optimization goal of the loss function.

The other is that once training is complete, we must use the output feature vectors to classify input road surface defect images. This involves designing an effective classifier based on the trained model to recognize and classify different classes of road surface defects based on feature vectors. This may include selecting suitable classification algorithms and determining how to handle and interpret classification results effectively in practical applications.

Additionally, considerations include how to train a classification model with good generalization performance using a small number of samples, and how to ensure the model can continuously learn new classes after deployment.

### 3.2. Overall Framework

Deep metric learning exhibits fundamental distinctions from traditional supervised learning in both training and testing paradigms. Conventional supervised learning typically employs loss functions such as cross-entropy or mean squared error to minimize discrepancies between predicted outputs and ground-truth labels, where each sample is represented by a one-hot encoded class vector. In contrast, deep metric learning focuses on learning task-specific distance or similarity metrics, utilizing pairwise or triplet-based loss functions that explicitly optimize the relative distances between samples in the embedding space. During inference, traditional supervised models directly predict class labels through forward propagation of test samples, while deep metric learning systems compute distances or similarities between test samples and reference data (e.g., class prototypes or training exemplars) to execute classification, clustering, or retrieval tasks. This divergence underscores their core philosophical differences: deep metric learning prioritizes relational structure learning between samples, whereas traditional supervised learning emphasizes precise alignment between model outputs and discrete labels. The methodology proposed in this work follows the deep metric learning paradigm, structured into three sequential phases—dataset preparation, model training, and model evaluation—each of which will be elaborated in subsequent sections.

### 3.3. Dataset Preparation

The UAV-PDD2023 dataset [27] is an open-source benchmark specifically designed for unmanned aerial vehicle (UAV)-based pavement distress detection. It contains 2439 three-channel JPG images covering six predefined distress categories: longitudinal cracks (LC), transverse cracks (TC), alligator cracks (AC), oblique cracks (OC), repair marks (RP), potholes (PH). The detailed analysis of the dataset can be found in the original UAV-PDD2023 publication. To conserve space, this paper does not provide an in-depth introduction. For this study, all distress instances were extracted as square regions from the original images and saved into category-specific folders. The resulting instance counts per distress type are summarized in Table 1. This preprocessing step ensures consistent input dimensions while preserving the critical spatial features of each distress pattern.

To train and evaluate the method based on deep metric learning proposed in this paper, five sample sets were constructed, corresponding to five separate folders. Among them, the training set, validation support set, and validation set are used during the model training phase, while the testing support set and testing set are used during the model evaluation phase, as shown in Table 2.

To simulate real-world application scenarios, this study includes the three most common disease categories (longitudinal cracks, transverse cracks, and diagonal cracks) in all five sample sets. In contrast, the three less common and rare disease categories (alligator cracking, potholes, and patch marks) are treated as online extension classes and are only included in the testing support set and the testing set.

The reason for separating common and rare types of pavement distress during training is to simulate real-world application scenarios. In practice, when training the model, only data of common distress types may be available. In such cases, a feature extractor can be trained using only this available data. After the model is deployed, if new types of distress are encountered, images of these new types can be captured and their features extracted using the pre-trained feature extractor. These extracted features can then be added to the support set. During the subsequent classification phase, the newly added distress types can automatically participate in the classification process without the need to retrain the feature extractor. This enables the proposed method to effectively adapt to new types of pavement distress.

The number of instances in the training set is shown in Table 3.

A total of 100 instances were sampled from each disease category in the training set to form the validation support set. The instance counts are presented in Table 4.

The validation set also contains the same three types of diseases as the training set, but there is no overlap in data between the two sets, as shown in Table 5.

The test support set contains six types of diseases, including all the samples used during the training phase, along with three additional types of rare (relatively less common) disease samples, as shown in Table 6.

The test set contains the same six types of diseases as the test support set, with no data overlap with the other four sample sets, as shown in Table 7.

### 3.4. Model Training

First, the BNInception model is built and combined with a feature encoding layer that maps feature maps into feature vectors, forming the final target model. Next, the SoftTriple loss function is constructed, which includes a trainable fully connected layer. After training, this layer implicitly contains the cluster centers for all classes.

The training dataset consists of three types of diseases with relatively large numbers of instances: 1892 images of longitudinal cracks, 1070 images of diagonal cracks, and 3443 images of transverse cracks.

During the model’s forward propagation, training is carried out using this dataset containing the three types of diseases. After the model reads the input images, it generates feature maps through the BNInception network, which are then passed through a feature encoding matrix to produce N-dimensional feature vectors.

The SoftTriple loss is calculated for these feature vectors as follows: the feature vector is passed through a linear layer in the SoftTriple function to compute the similarity between the feature vector of each image and all cluster centers. Since the SoftTriple loss allows each class to have multiple cluster centers, the similarities between the feature vector and all cluster centers of the same class are summed to obtain the total similarity for each class. Then, based on these similarities and the one-hot encoding of the ground truth labels, the classification loss is calculated. The objective of this loss is to maximize the similarity between the input image and the cluster centers of its true class (targeting a value of 1), while minimizing the similarity with other classes (targeting a value of 0).

After obtaining the classification loss, the regularization loss is calculated, which measures the similarity between different cluster centers. The goal of this regularization is to reduce inter-center similarity, thereby improving intra-class compactness and inter-class separability, ultimately enhancing the model’s classification performance. The total loss is the sum of the classification and regularization losses.

Finally, the model parameters are updated via backpropagation based on the computed total loss. After multiple iterations, a network model with strong feature encoding and classification performance can be obtained.

### 3.5. Model Testing

Since the trained model outputs only a feature vector for an input image and does not directly produce a classification result, it is necessary to design an additional category prediction method.

The goal of training a deep metric learning model is to ensure that images from the same class yield highly similar feature vectors, while images from different classes yield dissimilar ones. Therefore, after training, the model can estimate the class of an image with an unknown label by comparing the similarity between its feature vector and those of images with known labels. The image is then classified into the class whose images have the most similar features.

To achieve this, a support set containing representative images of all target classes is required. All images in this support set are first passed through the model to obtain their corresponding feature vectors. The image to be predicted is also passed through the model to obtain its feature vector. Then, the similarity between this vector and each vector in the support set is calculated. These similarities are sorted in descending order, and the classes of the top-ranked samples are considered more likely to match the class of the image being predicted.

The specific category prediction method is as follows: After ranking the support set images by their similarity to the query image, select the top k most similar samples. Count which classes are represented among these k samples. Then, for each class, sum the similarity scores of its samples among the k selected ones, and divide this sum by the total number of samples of that class in the support set. This result is taken as the score for that class. Finally, the class with the highest score is selected as the prediction result. The overall procedure is illustrated in Algorithm 1.
**Algorithm 1.** Category Prediction Method  **Input:** A list of labels y corresponding to support set samples, sorted in descending order of feature similarity with the query image; a list of similarity scores s, aligned with y; a hyperparameter k indicating the number of top similar samples to consider.  **Output:** Predicted label $y_{pred}$
  Define a function ${N(y}_{i})$ that returns the total number of support set samples belonging to class $y_{i}$.  Extract the top k elements from y and s to form labels and sims.  Initialize an empty dictionary socre_dict to store the cumulative score for each class, and set the initial score of each class to 0. for  i⟵1  to  k  do   $socre\_dict_{{labels}_{i}}⟵socre\_dict_{{labels}_{i}}+\frac{{sims}_{i}}{{N(labels}_{i})}$ endfor  Obtain the key in socre_dict with the maximum value as the predicted label $y_{pred}$. $return\;\;y_{pred}$


To alleviate the problem of class imbalance in the support set and to prevent majority-class samples from dominating the similarity-based prediction, we design a weighting strategy based on both similarity and class sample count:

(12) $$\omega_{i}=\frac{similarity(x,x_{i})}{N(y_{i})}$$

This strategy combines feature similarity with class density and has a reasonable theoretical foundation. From the perspective of representation learning, it incorporates a prior reflecting “sample sparsity” when weighting support samples within the same class. From a probabilistic modeling viewpoint, it resembles a soft voting mechanism within each class and implicitly treats each support sample as a potential sub-center. Moreover, this method is consistent with the multi-center concept in SoftTriple loss, enhancing the model’s ability to capture intra-class variation.

Through this design, the model can flexibly recognize all disease categories covered by the support set based on the sample information it provides. Even if some categories were not introduced during training, the model can dynamically adapt and perform classification as long as the support set contains examples of those categories—demonstrating an ability for online class-incremental learning. When a certain class is not represented in the support set, the proposed method assigns the query image to the most similar known class. To achieve accurate classification in such cases, it is necessary to include representative samples of that class in the support set.

## 4. Class-Incremental Comparison Experiments

### 4.1. Experimental Design

To test the online class-incremental ability of the proposed method for rare disease categories, this method (Control Group 1) is compared with the iCaRL class-incremental method (Control Group 2). Both the proposed method and the iCaRL method are trained using the same three common disease categories (including transverse cracks, vertical cracks, and diagonal cracks). In each training round, the validation set is used to validate the results of that round. During validation, there are slight differences between the proposed method and the iCaRL method, as the proposed method requires validation of the support set to assist in prediction, while the iCaRL method does not.

After the training phase, the iCaRL method requires incremental training for three additional rare disease categories, while the proposed method does not require any further training. Once the incremental training of the iCaRL method is complete, both methods are tested on the same test set, and the classification capabilities of both models are compared for six disease categories: transverse cracks, vertical cracks, diagonal cracks, crazing, repair marks, and potholes.

### 4.2. Experimental Environment and Parameter Settings

All experiments in this paper were conducted on a machine with an AMD Ryzen 7 6800U CPU, running Windows 11 as the operating system. The experimental environment includes Python 3.8.18 and PyTorch 1.8-cpu.

To meet the minimum input size requirement of the BNInception network, the input images were resized to 224 × 224 while maintaining their original aspect ratio. Any blank areas were filled with black color. The output feature vector dimension of the network’s fully connected layer was set to 64, and the feature vectors were standardized using the L2 norm [28].

The training parameters for both methods were the same. Adam [29] was used as the optimizer, with an initial learning rate of 0.0001 for the model. The initial learning rate for the SoftTriple cluster center fully connected layer was set to 0.01, with ε = 0.01 and weight decay set to 0.0001. The batch size during training was set to 32. The BN layer parameters were updated during training, while they were frozen during testing. The model was trained for a total of 50 epochs. The focus of this work lies in proposing an online class-incremental classification method tailored for pavement distress detection, rather than extensively exploring optimal parameter configurations for SoftTriple loss. We have adopted the commonly used parameter settings found in the literature to ensure stability and reproducibility.

### 4.3. Evaluation Metrics

The experiments use three commonly used image classification evaluation metrics to assess the model’s classification performance: Accuracy, Recall, and F1_score.

Accuracy is one of the key metrics for measuring the performance of a classifier. It represents the ratio of the number of correctly classified samples to the total number of samples. In simple terms, Accuracy measures the proportion of correctly classified samples out of all samples. The formula for calculating Accuracy is

(13) $$\begin{array}{c} Accuracy=\frac{TP+TN}{TP+TN+FP+FN} \end{array}$$

In the formula, TP represents true positive, TN represents true negative, FP represents false positive, and FN represents false negative.

Recall, also known as sensitivity or the true positive rate, measures the model’s ability to correctly identify positive class samples. Specifically, Recall refers to the proportion of actual positive samples that are correctly predicted as positive by the model. The formula for calculating Recall is

(14) $$\begin{array}{c} Recall=\frac{TP}{TP+FN} \end{array}$$

In the formula, TP represents true positive and FN represents false negative.

F1_score is a metric that combines both Precision and Recall to assess the performance of a classifier. The F1_score is the harmonic mean of these two metrics and can balance the trade-off between Precision and Recall. The formula for calculating the F1_score is

(15) $$\begin{array}{c} F1\_score=2\times \frac{Precision\times Recall}{Precision+Recall} \end{array}$$

where Precision represents the precision, which is calculated as

(16) $$\begin{array}{c} Precision=\frac{TP}{TP+FP} \end{array}$$

The F1_score ranges from 0 to 1, with higher values indicating a better classification performance of the model.

Since this paper addresses a multi-class classification problem, the average F1_score for all categories is calculated as the model’s overall classification performance metric. The average F1_score is typically calculated using either the macro-average or weighted average of the F1_score across all classes:

(17) $$\begin{array}{c} F{1\_score}_{mean}=\frac{\sum_{i=1}^{C}F1\_score_{i}}{C} \end{array}$$

In the formula, C represents the number of classes, and i represents the class index.

In addition to using the three metrics mentioned above to evaluate the various disease categories, additional metrics are required to assess the overall performance of the model. The macro-average and weighted average are two commonly used methods for aggregating multi-class metrics, which provide a comprehensive reflection of the model’s overall performance across all categories. These methods behave differently when addressing class imbalance issues.

The macro-average calculates the metric (such as Precision, Recall, or F1_score) for each class individually and then takes the arithmetic average across all classes. It does not take into account the number of samples, so each class has the same weight. For example, the calculation formula for $Macro_{Precision}$ is

(18) $$\begin{array}{c} Macro_{Precision}=\frac{Precision_{class1}+Precision_{class2}+\ldots +Precision_{classN}}{N} \end{array}$$

where N represents the total number of categories.

The macro-average treats each class equally. If there are classes with very few samples, their performance fluctuations can significantly affect the macro-average results. Therefore, the macro-average is suitable for scenarios where the importance of each class is the same.

The weighted average calculates the metric for each class individually and then computes a weighted sum based on the proportion of samples in each class. Categories with a larger number of samples have a greater influence on the final result. The calculation formula for $Weighted_{Precision}$ is

(19) $$\begin{array}{c} Weighted_{Precision}=\sum_{i=1}^{N}\left(Precision_{class_{i}}\times \frac{N_{class_{i}}}{N_{total}}\right) \end{array}$$

where $N_{class_{i}}$ is the number of samples in the class, $N_{total}$ is the total number of samples across all classes, $Precision_{class_{i}}$ represents the Precision for class i.

### 4.4. Training Procedure of Class-Incremental Comparison Experiments

For the proposed method, the BNInception model is trained for 50 epochs using a training set consisting of three common disease categories (vertical cracks, transverse cracks, and diagonal cracks). After each training epoch, the model’s classification ability is validated using the validation support set and the validation set. During the validation process, since the validation support set contains 300 images, we traverse the hyperparameter *k* in Algorithm 1 from 1 to 300 to observe its impact on Accuracy.

**Figure 2:**
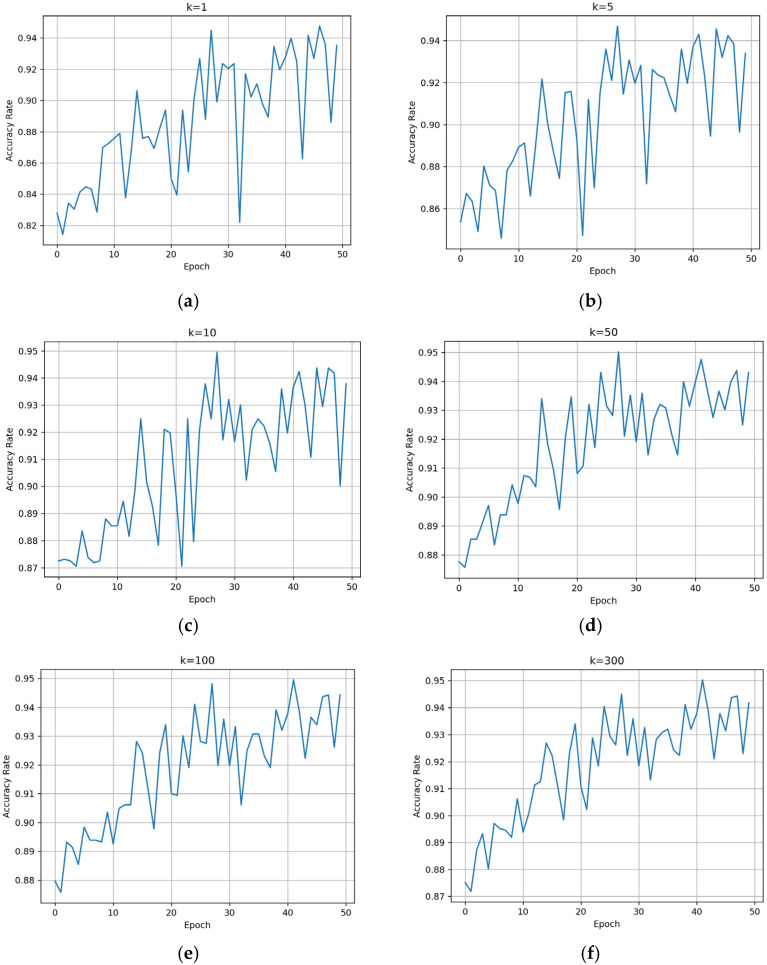
Accuracy variation with different values of *k* during training: (**a**) k = 1; (**b**) k = 5; (**c**) k = 10; (**d**) k = 50; (**e**) k = 100; (**f**) k = 300.

*k* values ranging from 1 to 300 are recorded. The resulting validation performance is shown in Figure 2.

**Figure 3:**
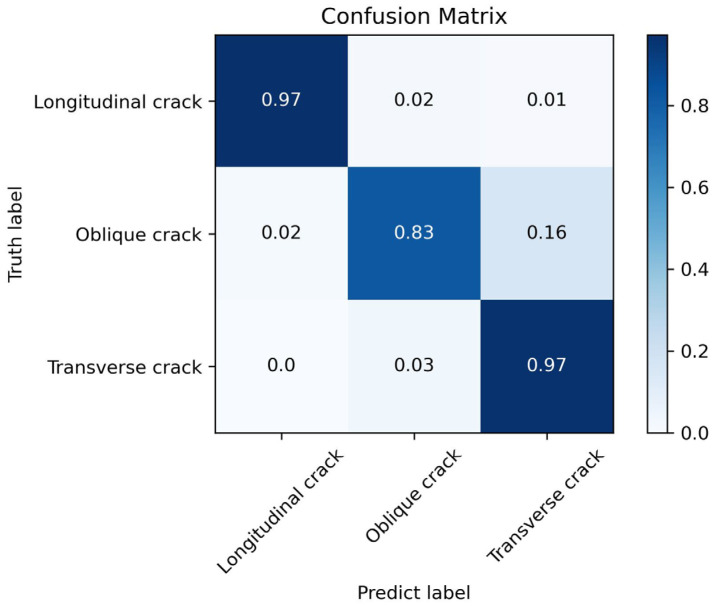
Confusion matrix of the proposed method on the validation set for the three disease categories.

It can be observed that as the number of training epochs increases, the Accuracy shows an overall upward trend. The larger the value of *k*, the more stable the Accuracy becomes. By the 50th training epoch, the Accuracy for the three disease categories reaches approximately 94%. The confusion matrix tested on the validation set is shown in Figure 3.

From the confusion matrix in Figure 3, it can be seen that the trained model exhibits a good classification ability for the three disease categories. At this point, the proposed method has completed its training.

**Figure 4:**
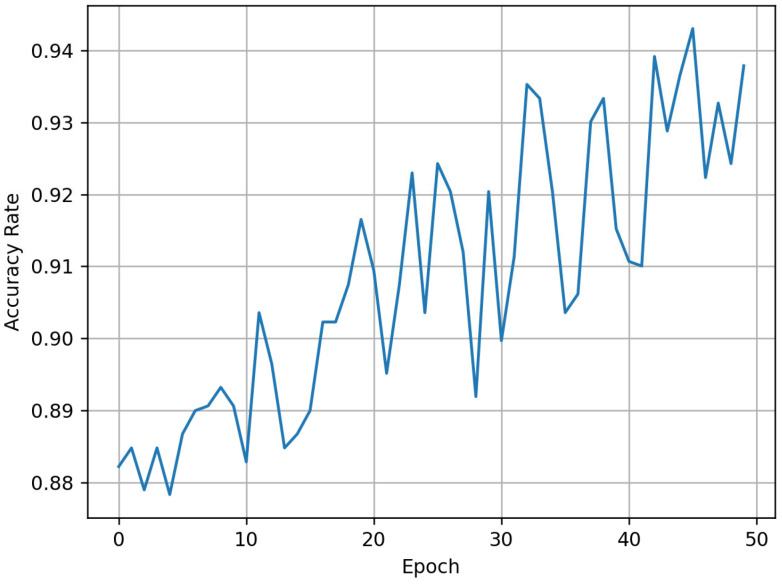
Accuracy variation during the first phase of training using the iCaRL method.

Next, the iCaRL method is used for the first phase of training. The model is initially trained using the same three common disease categories (transverse cracks, vertical cracks, and diagonal cracks) as the training set, with training parameters identical to those in control group 1. The Accuracy changes during the training process are shown in Figure 4.

**Figure 5:**
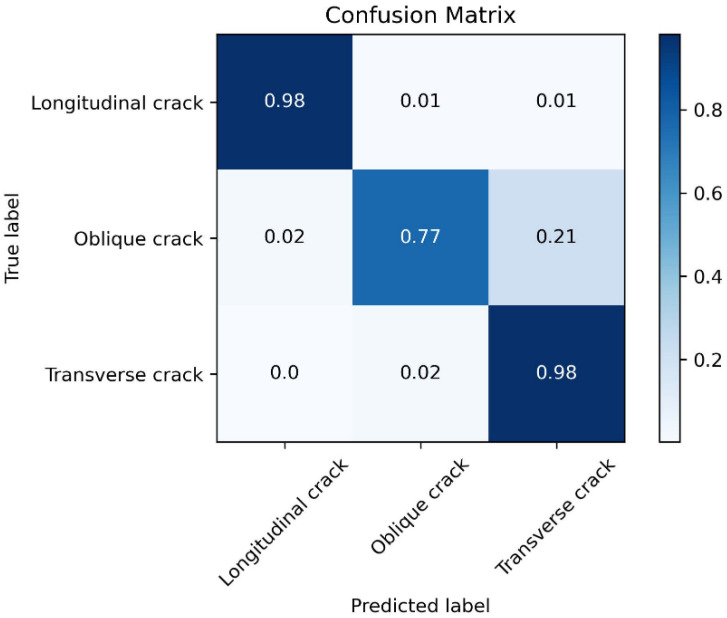
Confusion matrix of the iCaRL method after the first phase of training.

It can be observed that after the first phase of training using the iCaRL method, the model achieves an Accuracy of 94% for classifying the three common disease categories. The confusion matrix for this model is shown in Figure 5.

Next, the second phase of incremental class training is carried out using the iCaRL method, where three additional rare disease categories (cracking, repair traces, and potholes) are introduced. A total of 50 epochs are used for this second phase of training. After training is completed, both control groups have prepared models ready for testing.

### 4.5. Results of Class-Incremental Comparison Experiments

**Figure 6:**
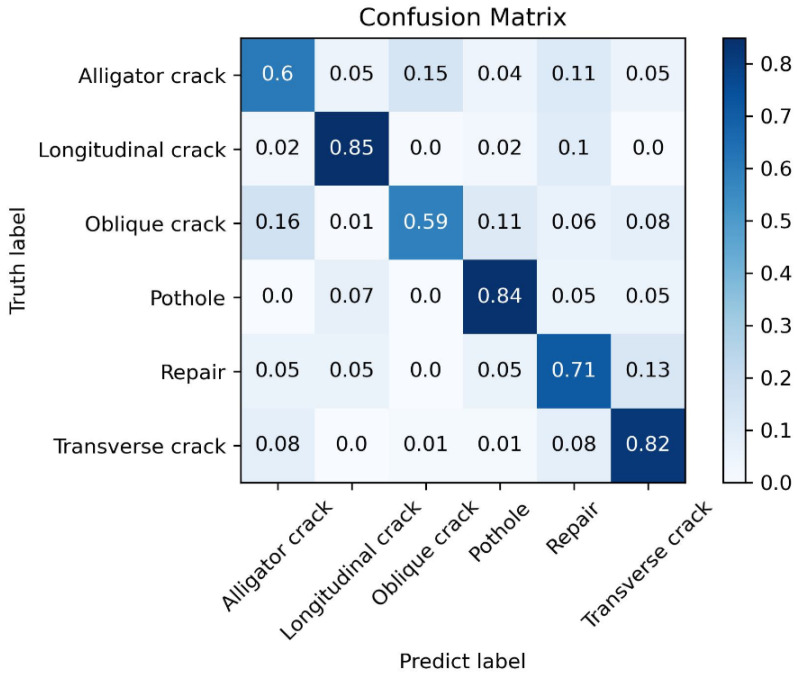
Confusion matrix of the proposed method on the test set.

**Figure 7:**
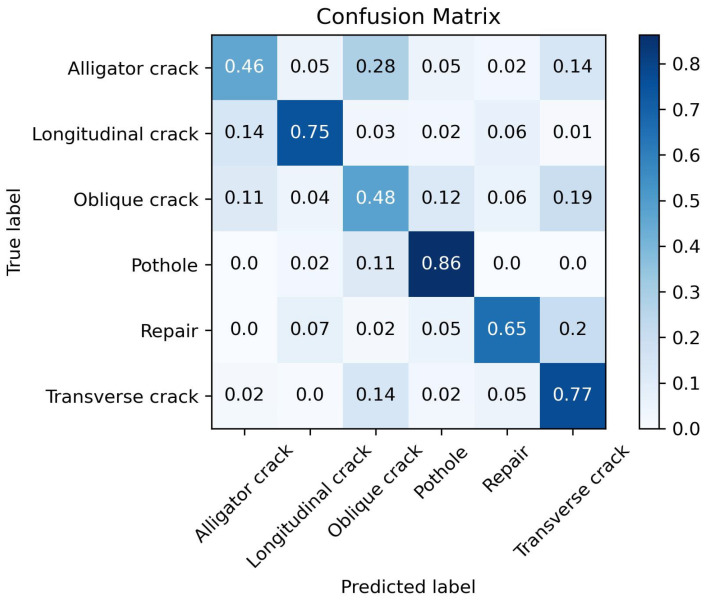
Confusion matrix of the iCaRL method on the test set.

The models from control group 1 and control group 2 are tested on the test set introduced in the previous section. The obtained test results are shown in Figure 6 and Figure 7 and Table 8 and Table 9.

From the experimental results, it can be seen that the macro-average F1_score and weighted average F1_score of the proposed method are 60.9% and 81.5%, respectively, which are 6.7% and 8.5% higher than those of the iCaRL method. This indicates that the proposed method demonstrates a better incremental class performance.

## 5. Traditional Supervised Learning Comparison Experiments

### 5.1. Experiment Design

To further investigate the classification accuracy performance of the proposed method (control group 1) compared to traditional supervised learning methods (control group 2), the following experiment is conducted. Both control groups are trained on the same training set, which includes all six disease categories, using the same training parameters as in the previous section. After training, the models are tested on the same test set containing all six disease categories, and the experimental results are compared.

### 5.2. Training Procedure of Traditional Supervised Learning Comparison Experiments

**Figure 8:**
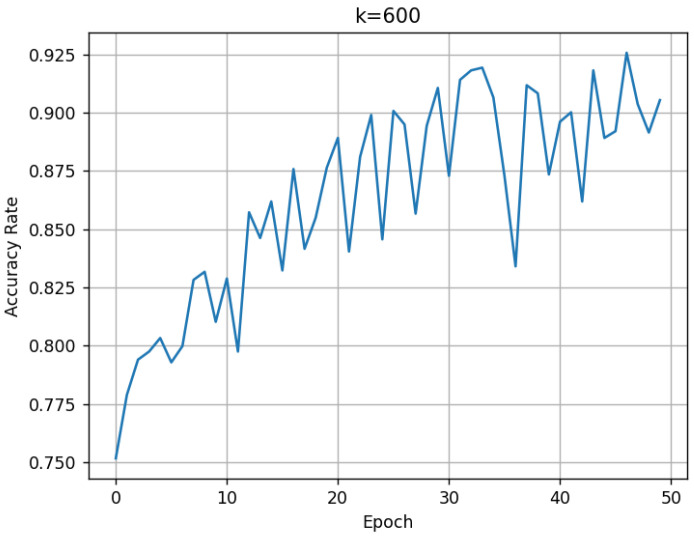
Accuracy variation in the proposed method during the training process.

**Figure 9:**
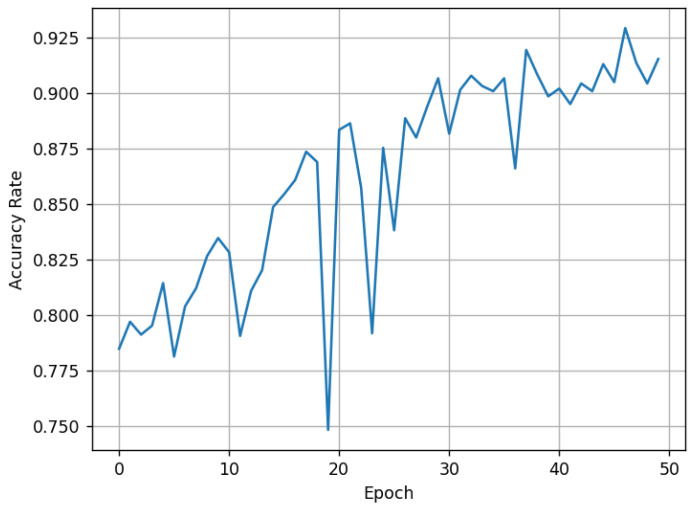
Accuracy variation in the traditional supervised learning method during the training process.

After each training epoch, both methods are validated using a validation set that includes all six disease categories. The Accuracy changes during training are shown in Figure 8 and Figure 9.

Both learning methods converge at an Accuracy of approximately 92.5%.

### 5.3. Results of Traditional Supervised Learning Comparison Experiments

**Figure 10:**
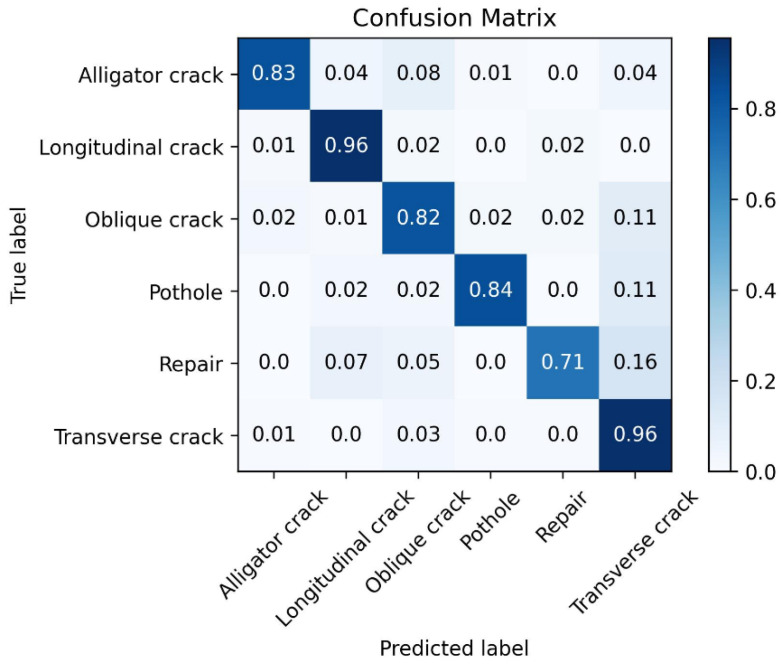
Confusion matrix of the proposed method.

**Figure 11:**
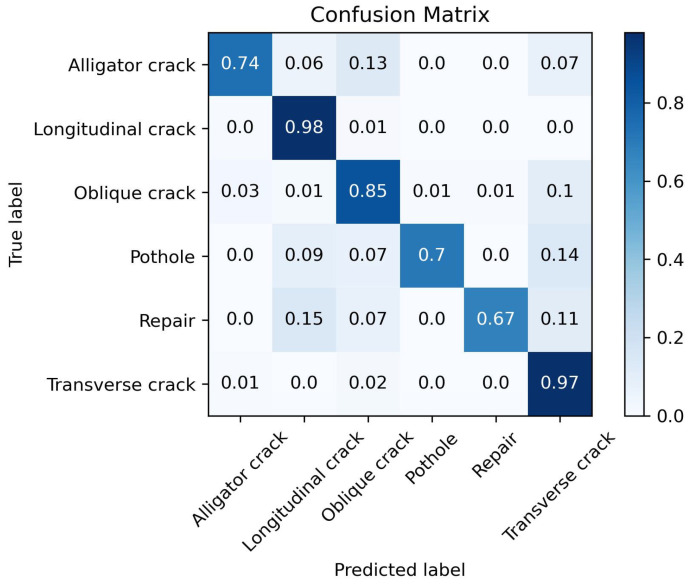
Confusion matrix of the traditional supervised learning method.

The models trained using both learning methods are tested on the same test set as in the previous section. The obtained confusion matrices and metrics are shown in Figure 10 and Figure 11 and Table 10 and Table 11.

From a comparison of the experimental results, it can be seen that the macro-average Recall of the proposed method is 85.1%, which is 3.2% higher than that of the traditional supervised learning method. The macro-average Precision is 82.5%, which is 7.6% lower than that of the traditional method. The difference in the macro-average F1_score is not significant. The proposed method is suitable for application scenarios where the Recall rate for pavement damage classification is more important.

## 6. Software Design

**Figure 12:**
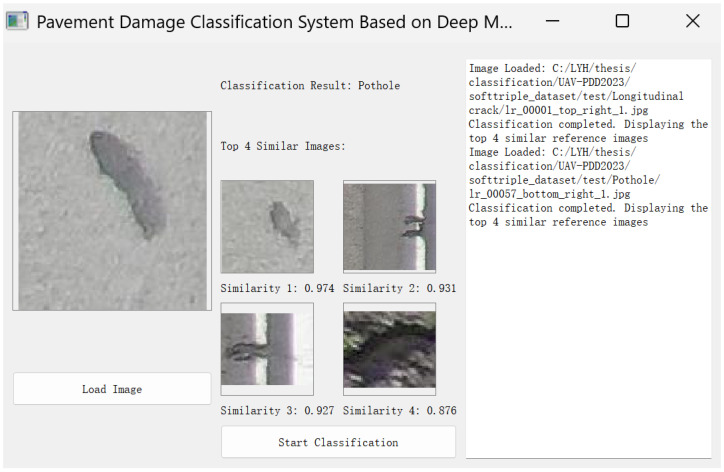
Software design.

The image classification software was designed based on the method described in this paper, with features such as loading images, classifying images, displaying classification results, and logging. It can also display the top four images most similar to the test image, as shown in Figure 12.

The software can effectively classify pavement damage images and enhance the interpretability of the classification results by displaying the top four images most similar to the test image from the support set.

## 7. Discussion

The proposed deep metric learning framework tackles two key difficulties in pavement distress classification: the need for large-scale annotated datasets and the inflexibility in handling new distress categories incrementally.By establishing multiple intra-class centroids through SoftTriple loss, our method explicitly models the high intra-class variance commonly observed in real-world distress images (e.g., cracks with varying orientations, scales, and lighting conditions), a challenge inadequately handled by conventional single-center supervised learning. This design aligns with recent findings in material defect detection, where multi-cluster representation improves feature discrimination for irregular patterns [30].

Our adaptive weighting strategy demonstrates superior performance over class-rebalancing techniques like Focal Loss [31], particularly in scenarios with an extreme data imbalance (e.g., rare distress types in newly constructed roads). Unlike SMOTE-based oversampling [32], which risks generating unrealistic synthetic samples, our approach leverages both sample similarity and class priors to dynamically adjust learning focus—proven effective by the 6.7% macro-F1 improvement over iCaRL. This echoes advancements in few-shot medical image analysis [33], where similarity-guided adaptation outperforms explicit data augmentation.

The soft-label mechanism provides a viable solution to labeling noise, a persistent issue in pavement inspection where visual similarities between distress types (e.g., alligator cracks vs. map cracks) often lead to annotation inconsistencies. By evaluating sample-to-exemplar similarity, our method achieves “self-cleaning” of noisy labels, analogous to prototype refinement strategies in semi-supervised metal surface defect detection [30]. However, unlike the work of [30] that requires iterative clustering, our single-pass support-set comparison reduces computational overhead—a critical advantage for UAV-based real-time inspection.

Practical deployment considerations reveal two promising directions: First, the method’s compatibility with incremental learning makes it adaptable to region-specific distress patterns (e.g., freeze-thaw cracks in cold climates vs. thermal cracks in deserts), enabling localized model updates without full retraining. Second, the reduced annotation dependency aligns with industry needs for low-cost pavement monitoring, particularly in developing regions with limited inspection budgets.

Key limitations include the following: (1) performance degradation when novel classes exhibit visual overlaps with multiple existing clusters, a scenario requiring explicit cross-centroid relationship modeling; (2) dependence on UAV image quality—heavy shadows or occlusions may distort similarity measurements, necessitating fusion with LiDAR or infrared data in future work.

Inference speed depends on various factors, including hardware capabilities, the structure of the feature extractor, and the size of the support set. This work focuses on proposing an online class-incremental classification method tailored to pavement distress, rather than practical deployment. In real-world applications, one can select more powerful hardware, lightweight feature extractors, and an appropriately sized support set to increase the FPS.

## 8. Conclusions

This chapter addresses the issues of traditional convolutional neural network (CNN) learning methods in road damage image classification tasks, such as low accuracy, limited sample sizes for rare damage categories, and the need to retrain the model when new damage categories are introduced. To overcome these challenges, a deep metric learning approach is innovatively introduced for pavement damage classification research. The SoftTriple loss function is used to train the BNInception network, enabling the network’s output feature vectors to exhibit strong intra-class cohesion and inter-class separation. Then, similarity calculations are performed with the features of the exemplar images in the support set, and the final predicted category is determined using a category prediction strategy.

The experimental results show that the classification method proposed in this chapter can train a network model with good classification performance, even with limited samples for rare damage categories. Its macro-average Recall is 3.2% higher than traditional supervised learning methods, making it suitable for application scenarios where high Recall rates are critical for pavement damage classification. Moreover, the method has online class-incremental capabilities. A comparison with the iCaRL incremental learning method reveals that the proposed method achieves a 6.7% and 8.5% increase in the macro-average F1_score and weighted average F1_score, respectively, demonstrating a better classification performance for newly added rare damage categories. The method is both practical and scalable.

## Figures and Tables

**Table 1:** Instances per disease category.

Disease Types	Quantities
Alligator cracks	603
Longitudinal crack	2994
Oblique crack	1686
Pothole	195
Repair	282
Transverse crack	5394

**Table 2:** The five sample sets.

Folder Name	Set Name	Usage Phase
train	Training Set	Training
val_support	Validation Support Set	Training
val	Validation Set	Training
test_support	Testing Support Set	Testing
test	Testing Set	Testing

**Table 3:** Number of instances in the training set.

Disease Type	Quantity
Longitudinal crack	1892
Oblique crack	1070
Transverse crack	3443

**Table 4:** Number of Instances in the Validation Support Set.

Disease Type	Quantity
Longitudinal crack	100
Oblique crack	100
Transverse crack	100

**Table 5:** Number of instances in the validation set.

Disease Type	Quantity
Longitudinal crack	451
Oblique crack	263
Transverse crack	831

**Table 6:** Number of instances in the test support set.

Disease Type	Quantity
Alligator crack	380
Longitudinal crack	1892
Oblique crack	1070
Pothole	115
Repair	184
Transverse crack	3443

**Table 7:** Number of instances in the test set.

Disease Type	Quantity
Alligator crack	124
Longitudinal crack	651
Oblique crack	353
Pothole	44
Repair	55
Transverse crack	1120

**Table 8:** Test results of the proposed method in class-incremental comparison experiments.

Disease Type	Precision	Recall	F1-Score	Support
Alligator crack	0.316456	0.604839	0.415512	124
Longitudinal crack	0.971880	0.849462	0.906557	651
Oblique crack	0.855372	0.586402	0.695798	353
Pothole	0.330357	0.840909	0.474359	44
Repair	0.174888	0.709091	0.280576	55
Transverse crack	0.954357	0.821429	0.882917	1120
Macro Average	0.600552	0.735355	0.609287	2347
Weighted Average	0.880663	0.780145	0.814862	2347

**Table 9:** Test results of the iCaRL method in class-incremental comparison experiments.

Disease Type	Precision	Recall	F1-Score	Support
Alligator crack	0.27804878	0.459677419	0.346504559	124
Longitudinal crack	0.941972921	0.748079877	0.83390411	651
Oblique crack	0.443569554	0.478753541	0.460490463	353
Pothole	0.292307692	0.863636364	0.436781609	44
Repair	0.236842105	0.654545455	0.347826087	55
Transverse crack	0.895010395	0.76875	0.827089337	1120
Macro Average	0.514625241	0.662240443	0.542099361	2347
Weighted Average	0.780818905	0.702172987	0.72990207	2347

**Table 10:** Test results of the proposed method in traditional supervised learning comparison experiments.

Disease Type	Precision	Recall	F1-score	Support
Alligator crack	0.844262	0.830645	0.837398	124
Longitudinal crack	0.977987	0.955453	0.966589	651
Oblique crack	0.835735	0.82153	0.828571	353
Pothole	0.711538	0.840909	0.770833	44
Repair	0.629032	0.709091	0.666667	55
Transverse crack	0.948582	0.955357	0.951957	1120
Macro-average	0.824523	0.852164	0.837003	2347
Weighted average	0.922321	0.92075	0.921324	2347

**Table 11:** Test results of the traditional supervised learning method in traditional supervised learning comparison experiments.

Disease Type	Precision	Recall	F1-score	Support
Alligator crack	0.836364	0.741935	0.786325	124
Longitudinal crack	0.962293	0.980031	0.971081	651
Oblique crack	0.859195	0.847025	0.853067	353
Pothole	0.939394	0.704545	0.805195	44
Repair	0.860465	0.672727	0.755102	55
Transverse crack	0.948696	0.974107	0.961233	1120
Macro-average	0.901068	0.820062	0.855334	2347
Weighted average	0.930829	0.932254	0.930699	2347

## Data Availability

The data presented in this study are available on request from the corresponding author.
